# Fitness-related physical activity intensity explains most of the association between accelerometer data and cardiometabolic health in persons 50–64 years old

**DOI:** 10.1136/bjsports-2023-107451

**Published:** 2024-07-12

**Authors:** Jonatan Fridolfsson, Elin Ekblom-Bak, Örjan Ekblom, Göran Bergström, Daniel Arvidsson, Mats Börjesson

**Affiliations:** 1 Center for Health and Performance, Department of Food and Nutrition and Sport Science, University of Gothenburg, Gothenburg, Sweden; 2 Center for Lifestyle Intervention, Sahlgrenska University Hospital, Gothenburg, Sweden; 3 Department of Physical Activity and Health, Swedish School of Sport and Health Sciences GIH, Stockholm, Sweden; 4 Department of Molecular and Clinical Medicine, University of Gothenburg Sahlgrenska Academy, Gothenburg, Sweden; 5 Department of Clinical Physiology, Västra Götalandsregionen, Gothenburg, Sweden; 6 Center for Lifestyle Intervention, Department of Molecular and Clinical Medicine, University of Gothenburg Sahlgrenska Academy, Gothenburg, Sweden; 7 Sahlgrenska University Hospital, Gothenburg, Region Västra Götaland, Sweden

**Keywords:** Physical activity, Cardiovascular Diseases, Epidemiology, Public health

## Abstract

**ABSTRACT:**

**Objectives:**

To investigate the physical activity (PA) intensity associated with cardiometabolic health when considering the mediating role of cardiorespiratory fitness (CRF).

**Methods:**

A subsample of males and females aged 50–64 years from the cross-sectional Swedish CArdioPulmonary bioImage Study was investigated. PA was measured by accelerometry and CRF by a submaximal cycle test. Cardiometabolic risk factors, including waist circumference, systolic blood pressure, high-density lipoprotein, triglycerides and glycated haemoglobin, were combined to a composite score. A mediation model by partial least squares structural equation modelling was used to analyse the role of CRF in the association between PA and the composite score.

**Results:**

The cohort included 4185 persons (51.9% female) with a mean age of 57.2 years. CRF mediated 82% of the association between PA and the composite score. The analysis of PA patterns revealed that moderate intensity PA explained most of the variation in the composite score, while vigorous intensity PA explained most of the variation in CRF. When including both PA and CRF as predictors of the composite score, the importance of vigorous intensity increased.

**Conclusion:**

The highly interconnected role of CRF in the association between PA and cardiometabolic health suggests limited direct effects of PA on cardiometabolic health beyond its impact on CRF. The findings highlight the importance of sufficient PA intensity for the association with CRF, which in turn is linked to better cardiometabolic health.

**WHAT IS ALREADY KNOWN ON THIS TOPIC**

- The complex relationship between physical activity (PA), cardiorespiratory fitness (CRF) and health has led to conflicting findings on the interconnected role of CRF in the association between PA and cardiometabolic health.

**WHAT THIS STUDY ADDS**

- This study demonstrates that CRF plays a major interconnected role in the association between PA and a cardiometabolic health composite score, with 82% of the association being mediated through CRF.
- The study highlights the importance of sufficient intensity of PA, with vigorous intensity activities showing stronger associations with cardiometabolic health when the interplay with CRF is considered.

**HOW THIS STUDY MIGHT AFFECT RESEARCH, PRACTICE OR POLICY**

- CRF measurements capture the majority of information about health-beneficial PA, indicating that CRF can serve as a proxy for sufficient volume and intensity of PA for health benefits.
- In clinical settings, prescribing PA of sufficient intensity is important as it serves as a key factor in the association with CRF, which conveys the main health benefits of PA. PA of lower intensity might not be sufficient for substantial health benefits.

## Introduction

 The relationship between physical activity (PA), cardiorespiratory fitness (CRF) and cardiometabolic health is complex.^1^ Strong evidence suggests that both PA and CRF are associated with better cardiometabolic health when not controlling for each other.^1^,^3^ The positive association between PA and CRF is also well established.^4^ In addition, the association between PA and cardiometabolic health seems to be both moderated and mediated by CRF.^1^ Moderation is apparent by the dose–response relationship between PA and cardiometabolic health being steeper among low-fit individuals compared with individuals with high CRF.^5 6^ However, the current research regarding CRF as a mediator of the association between PA and cardiometabolic health is conflicting. The mediating role of CRF has been suggested to be a plausible physiological mechanism for the cardiometabolic health benefits of vigorous intensity PA.^7^ Understanding the health benefits from different PA intensities and the interconnected role of CRF is important for tailoring recommendations on PA in clinical and public health settings.^7 8^

Three criteria must be fulfilled to consider CRF a mediator: both PA and CRF must be associated with cardiometabolic health, PA and CRF must be associated with each other, and when both are predictors, CRF must dominate the association.^9^ Previous research conflicts on whether CRF dominates the relationship with cardiometabolic health or not. Some studies suggest that the association between PA and cardiometabolic health is fully mediated,^10^,^14^ or mediated to a large degree through CRF.^15 16^ Other studies suggest that PA and CRF are independently associated with cardiovascular risk.^17^,^19^ There are also results suggesting that only CRF,^20^ or only PA,^21^ is associated with cardiometabolic health even when not controlling for the other. Inconsistencies in previous research may be due to diverse measurement methods used for estimating PA and CRF. Measurement of PA is particularly different and is based on self-report,^10 11 13 14 16 18^ accelerometry,^15 17 19 20^ heart rate^21^ or a multisensory armband.^12^ All of these methods result in course measures representing either time spent at moderate-to-vigorous intensity PA or total energy expenditure. Recent methodological advancements have improved the measurement of PA intensity from accelerometers,^22^ and more sophisticated statistical approaches have enabled detailed analyses of health-beneficial PA intensity.^22 23^

The aim of this study was to investigate the PA intensity associated with cardiometabolic health when considering the mediating role of CRF.

## Methods

### Study population

This study investigated a subsample of the Swedish CArdioPulmonary bioImage Study (SCAPIS), a nationwide multicentre study including 30 154 randomly selected males and females aged 50–64.^24^ The subsample consisted of 4185 individuals from one study centre where CRF measurement was conducted. These individuals provided complete data on cardiometabolic risk factors, CRF and PA. Clinical variables and CRF were measured on two or three separate occasions within 2 weeks, while PA was measured between two of these occasions. The data collection was carried out in 2013–2018. The study has been approved by the ethics committee at Umeå University (no. 2021-228-31M) and by the Regional ethical board in Gothenburg (no. 638-16). Written, informed consent was retrieved from all participants. Patients or the public were not involved in the design, conduct or evaluation of the study.

### Cardiometabolic risk factor composite score

The cardiometabolic risk factors analysed were waist circumference, systolic blood pressure (SBP), total cholesterol to high-density lipoprotein ratio (TC:HDL), triglycerides and glycated haemoglobin (HbA1c). These variables reflect central obesity, hypertension, dyslipidaemia and hyperglycaemia, and their clustering indicates cardiometabolic risk.^25 26^ Waist circumference was measured using standardised methods with a measuring tape.^27^ Fasting venous blood samples were collected to determine levels of HDL, triglycerides and HbA1c. SBP was measured twice in each arm using an automated device (Omron M10-IT, Omron Health care Co, Kyoto, Japan), and the mean of the measurements was used.

To create a composite score (CS) representing cardiometabolic health, the variables were combined by calculating the mean of the Z-score standardised variables. To ensure an equal influence of dyslipidaemia as central obesity, hypertension and hyperglycaemia in the CS, the mean of Z-score standardised TC:HDL and triglycerides was calculated first. Then, the CS was obtained by taking the mean of the Z-scores of waist circumference, HbA1c, SBP and the mean value of TC:HDL and triglycerides, for each participant. Finally, the CS was reversed (multiplied by −1) so that a positive value represents better cardiometabolic health.

### Cardiorespiratory fitness

CRF was estimated from the submaximal Ekblom-Bak cycle ergometer test.^28^ The test considers the heart rate response from two subsequent submaximal workloads and has high validity as reference to direct measurement (R^2^adj=0.91, SE of estimate: 0.28 L/min). Exclusion criteria were ongoing infections, known unstable cardiovascular disease, indication of cardiac disease by electrocardiography, medication with beta-blockers, weight above 125 kg or resting heartrate above 100 beats per minute.

### Physical activity

PA was measured by triaxial accelerometers (ActiGraph model GT3X+, wGT3X+or wGT3X-BT, ActiGraph, Pensacola, Florida, USA). Participants wore the accelerometer in an elastic belt over their right hip for seven consecutive days and were instructed to take it off when sleeping and during water-based activities. Raw accelerometer data from each axis were extracted and processed using a 0.29–4 Hz bandpass filter and combined to a vector magnitude.^29^ This method has been shown to better capture moderate-to-vigorous (MVPA) intensity PA compared with the most commonly used method of ActiGraph counts.^29 30^ An epoch length of 1 s was used. Non-wear time was defined as at least 60 min of zero output, with the allowance of up to 2 min of output below the sedentary threshold.^31^ A valid day was defined as at least 10 hours of wear-time and a valid measurement as at least four valid days.^32^

To enable a detailed analysis of PA intensity, processed accelerometer output was divided into a spectrum representing time spent at different intensities. The edges dividing the intensity spectrum variables were 0, 25, 50, 100 mg and so forth, increasing by 50 mg until 1 000 mg and above. The upper limit was chosen because more than 30% of individuals were missing data above this intensity. Non-wear time was also included in the analyses of PA patterns. In addition, previously calibrated crude cut-points were applied for reference.^29^ Accelerometer data processing was performed using MATLAB V.2022b (MathWorks, Natick, MA, USA).

### Statistical analyses

Traditional statistical methods encounter difficulties when analysing PA intensity spectrum variables due to their collinearity.^23^ The collinearity is caused by the closed structure of the PA variables representing a 24-hour movement behaviour and the similarity of PA variables representing neighbouring intensities. Multivariate pattern analysis, specifically PLS, has been successful in addressing this issue and examining the association between detailed PA intensity and health outcomes.^2223 33^,^35^ PLS identifies latent variables that are linear combinations of PA intensity variables, maximising their covariance with the outcome (in this case, CS). However, previous studies applying more extensive PLS models investigating confounding effects and potential mediators, such as CRF, cannot be interpreted as in traditional regression based statistics.^22 35 36^

To overcome these limitations and facilitate interpretation, the present study introduces PLS structural equation modelling (PLS-SEM). PLS-SEM is widely used in social sciences for comparing different theoretical models by testing how well they fit the data, as well as studying unobservable constructs that are measured indirectly.^37^ In this study, PLS-SEM was employed in a simplified manner to handle the collinearity of PA variables by treating them as a latent construct. PLS-SEM combines PLS’s latent variable calculation with path modelling, allowing for more complex models.^37^ The results obtained from PLS-SEM and PLS regression are essentially the same for the association between a set of PA intensity variables and a single-health outcome variable. However, PLS-SEM provides a more intuitive interpretation of covariates in the association between PA and health, as well as the ability to examine mediation through CRF.

**Figure 1:**
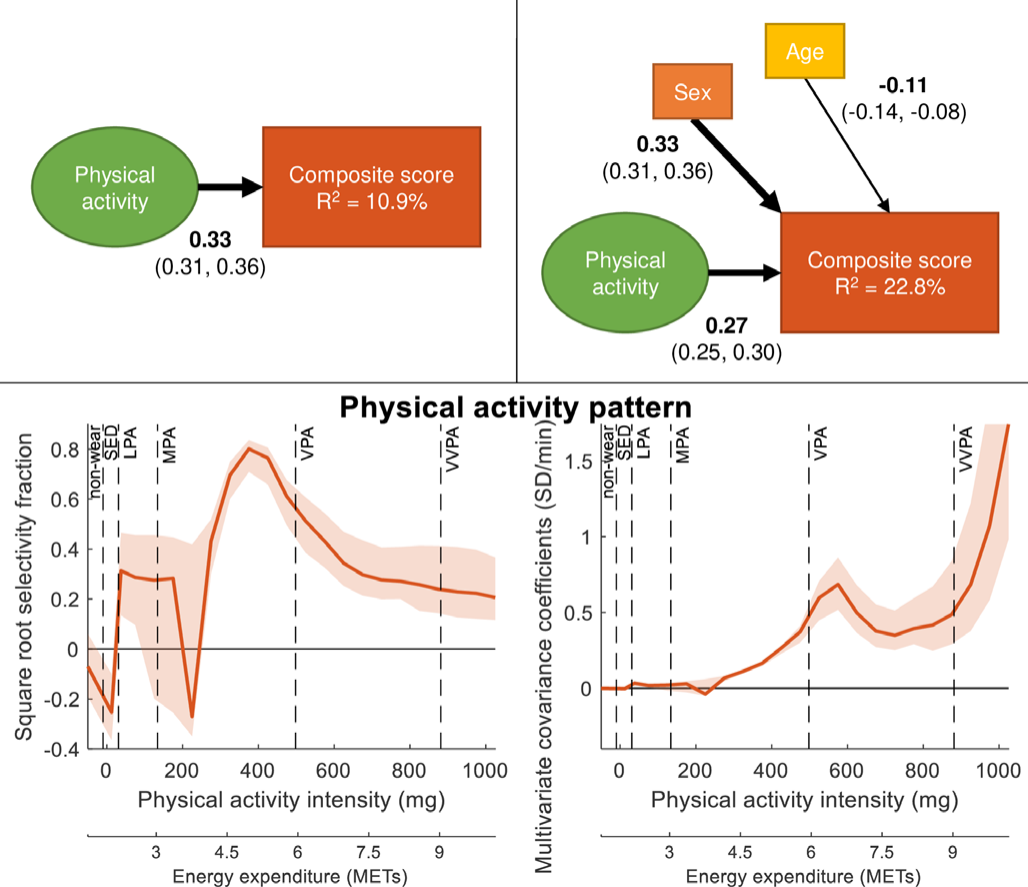
PLS-SEM models of the association between PA and CS, without (upper left) and with (upper right) controlling for sex and age. Path coefficients are shown together with 95% CIs. Boxes and circles represent observed and latent variables, respectively. The PA intensity pattern found to maximise the association with CS is shown standardised (bottom left) and unstandardised (bottom right) together with 95% CIs. Square root selectivity fraction between the PA intensity variables and the higher order PA construct represent the standardised contribution of different PA intensities in the latent variable representing PA. Multivariate covariance coefficients represent the unstandardised PA pattern of the different intensities in the latent PA variable. Non-wear time mainly consist of sleep time since participants were instructed to take the accelerometer off during sleep. CS, composite score; LPA, light intensity; METs, metabolic equivalents of task; MPA, moderate intensity; PA, physical activity; PLS-SEM, partial least squares structural equation modelling; SED, sedentary; VPA, vigorous intensity physical activity; VVPA, very-vigorous intensity physical activity.

The statistical concept of mediation was applied to study the interconnected role of CRF in the association between PA and CS. Seven different PLS-SEM models were set up with different structural models. First, a simple PLS model of the association between PA and CS. Second, a model of the association between PA and CS while controlling for sex and age. Third and fourth, models of the association between PA and CRF with and without controlling for sex and age. Fifth, a model of the association between CRF and CS while controlling for age and sex. Sixth, a model of the association between PA and CS with mediation through CRF while controlling CRF and CS for sex and age. Seventh, the same as model six but with the inclusion of an interaction term between sex and CRF to investigate the potential moderation effect in the association with CS. The structures of all models except number five and seven are displayed in figures1^3^. The proportion of the association that was mediated through CRF was calculated as the indirect association divided by the total association between PA and CS.^9 37^ The indirect association between PA and CS through CRF was calculated by multiplying the path coefficient between PA and CRF by the path coefficient between CRF and CS. The total association was the sum of the direct and indirect associations between PA and CS.

**Figure 2:**
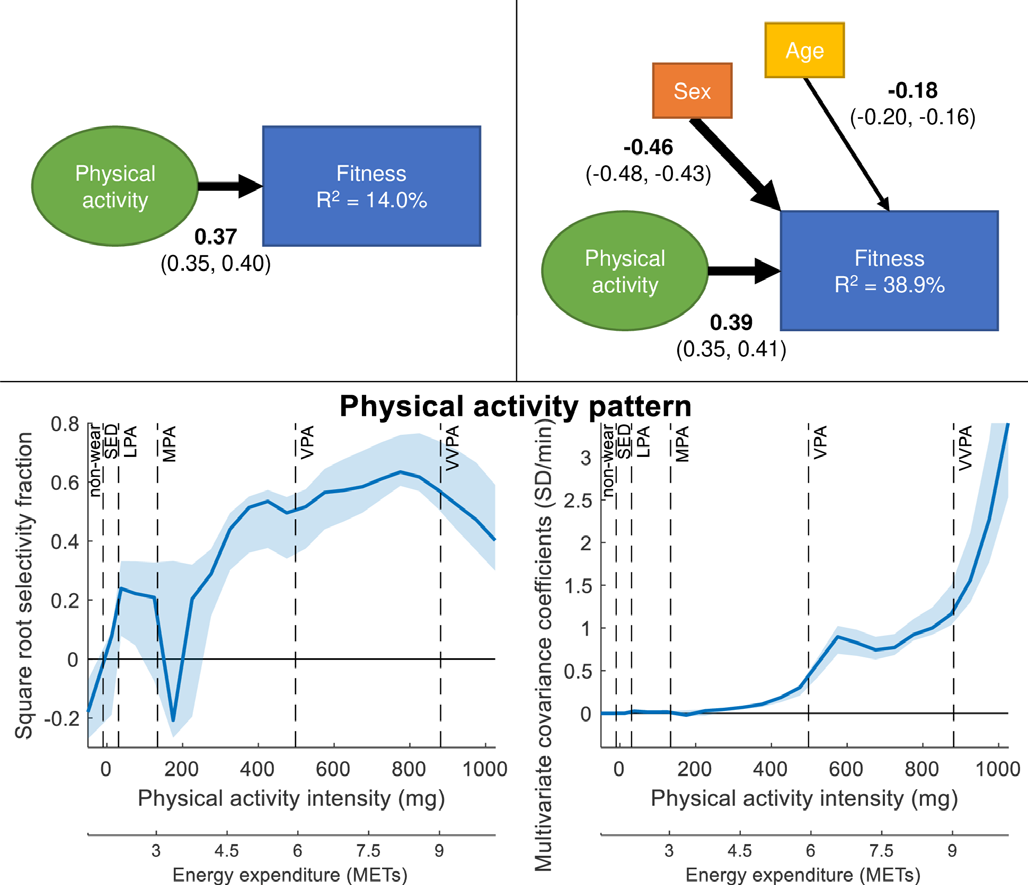
PLS-SEM models of the association between PA and CRF, without (upper left) and with (upper right) controlling for sex and age. Path coefficients are shown together with 95% CIs. Boxes and circles represent observed and latent variables, respectively. The PA intensity pattern found to maximise the association with CRF is shown standardised (bottom left) and unstandardised (bottom right) together with 95% CIs. Square root selectivity fraction between the PA intensity variables and the higher order PA construct represent the standardised contribution of different PA intensities in the latent variable representing PA. Multivariate covariance coefficients represent the unstandardised PA pattern of the different intensities in the latent PA variable. Non-wear time mainly consist of sleep time since participants were instructed to take the accelerometer off during sleep. CS, composite score; LPA, light intensity; METs, metabolic equivalents of task; MPA, moderate intensity; PA, physical activity; PLS-SEM, partial least squares structural equation modelling; SED, sedentary; VPA, vigorous intensity physical activity; VVPA, very-vigorous intensity physical activity.

**Figure 3:**
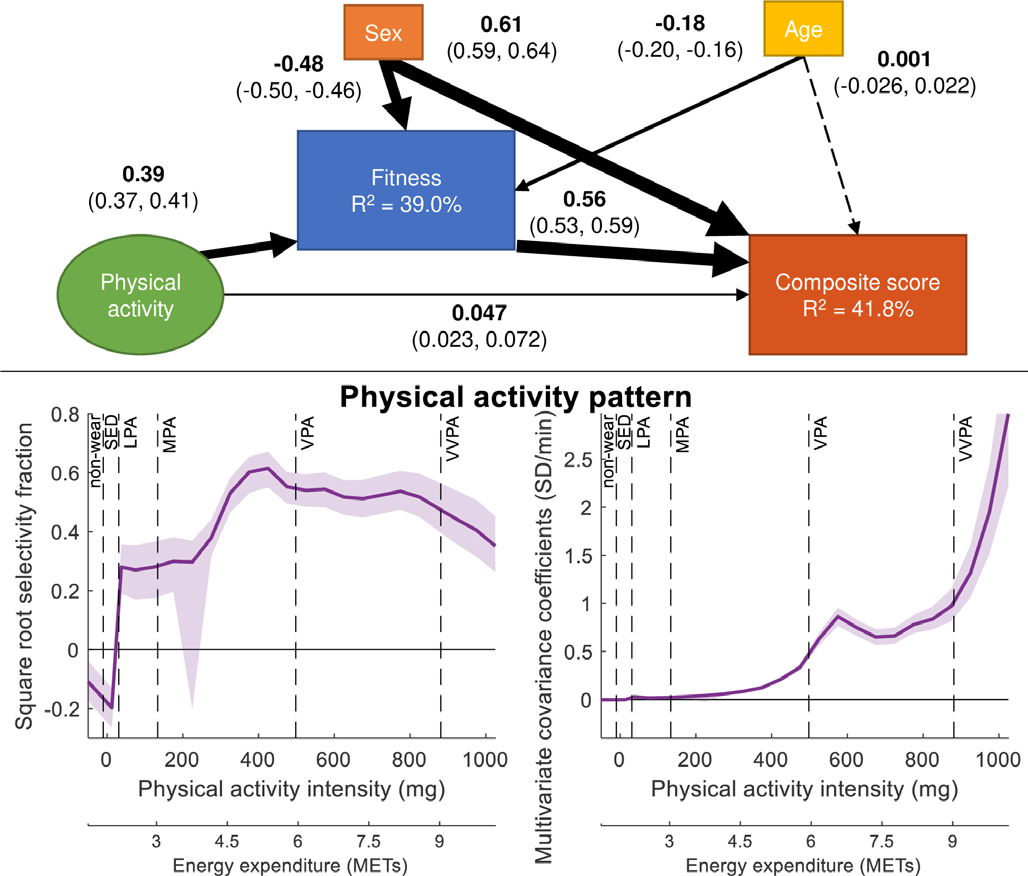
PLS-SEM model of the association between PA and CS and the mediation through CRF. CRF and CS are controlled for sex and age. Path coefficients are shown together with 95% CIs. Boxes and circles represent observed and latent variables, respectively. The PA intensity pattern found to maximise the association with CS is shown standardised (bottom left) and unstandardised (bottom right) together with 95% CIs. Square root selectivity fraction between the PA intensity variables and the higher order PA construct represent the standardised contribution of different PA intensities in the latent variable representing PA. Multivariate covariance coefficients represent the unstandardised PA pattern of the different intensities in the latent PA variable. Non-wear time mainly consist of sleep time since participants were instructed to take the accelerometer off during sleep. CS, composite score; LPA, light intensity; METs, metabolic equivalents of task; MPA, moderate intensity; PA, physical activity; PLS-SEM, partial least squares structural equation modelling; SED, sedentary; VPA, vigorous intensity physical activity; VVPA, very-vigorous intensity physical activity.

The PA intensity variables were represented by a latent variable specified as a higher order composite in the PLS-SEM models. Therefore, the PA intensity variables associations were not independent of each other and should be interpreted as an overall pattern. However, all variables in the structural model, including the latent PA variable, are independent and interpreted like in traditional multiple linear regression. The influence of the underlying PA intensity variables in the higher order composite representing PA was indicated by square root selectivity fraction (standardised) and multivariate covariance coefficients (unstandardised) of the association between the PA intensity variables and the higher order PA composite.^23 36^ Details regarding the specification of the PLS-SEM models are available in the online supplemental material. The statistical analyses were performed using R Statistical Software (V.4.1.2; R Core Team 2022) and the seminr package (V.2.3.2; Ray S, Danks N, Valdez A 2022).^38^

### Equity, diversity and inclusion statement

The SCAPIS study includes a random sample of the Swedish population to capture diversity in terms of sex, ethnicity, geography and socioeconomic status. Yet, the results might not be generalisable beyond the age range of the sample. The author team consists of junior, mid-career and senior researchers from different disciplines. However, the team is not fully gender-balanced and all authors are from the same country.

## Results

Table 1 presents the characteristics of the study sample, including males and females, with no significant age difference between the sexes. Females generally displayed more favourable health characteristics except for HbA1c, sleep and vigorous intensity PA.

**Table 1:** Study sample characteristics

	All	Male	Female
n (%)	4185 (100.0%)	2020 (48.3%)	2165 (51.9%)
Age (years)	57.2 (4.3)	57.2 (4.3)	57.1 (4.3)
Body mass index (kg∙m^2^)	26.4 (4.0)	26.9 (3.4)	25.9 (4.4)*
Waist circumference (cm)	92.4 (11.9)	97.7 (9.7)	87.5 (11.7)*
Systolic blood pressure (mm Hg)	121 (16)	125 (14)	117 (17)*
Glycated haemoglobin (mmol/mol)	35.0 (5.0)	35.1 (5.1)	35.0 (4.9)
Total cholesterol (mmol/L)	5.59 (1.02)	5.46 (1.01)	5.72 (1.01)*
High density lipoprotein (mmol/L)	1.72 (0.52)	1.48 (0.41)	1.93 (0.51)*
Triglycerides (mmol/L)	1.17 (0.97)	1.36 (1.27)	1.00 (0.50)*
Cardiorespiratory fitness (mL/min/kg)	33.9 (6.7)	36.9 (5.9)	31.1 (6.1)*
Physical activity			
Non-wear, including sleep (min/day)	556 (96)	553 (99)	558 (92)
Sedentary (min/day)	690 (99)	699 (101)	681 (96)*
Light (min/day)	106 (31)	103 (31)	109 (31)*
Moderate (min/day)	86.4 (28.3)	81.8 (28.6)	90.6 (27.3)*
Vigorous (min/day)	1.95 (3.92)	2.04 (4.27)	1.87 (3.57)
Very-vigorous (min/day)	0.19 (0.89)	0.23 (1.06)	0.15 (0.68)*

Mean (Standard deviationSD). * Significant sex difference with p from a two-sample t-test.

* Significant sex difference with p<0.05 from a two-sample t-test.

Figure 1 shows the two initial PLS-SEM models of the association between PA and CS without considering CRF. PA was associated with CS, slightly attenuated by controlling for sex and age. Male sex and older age were associated with lower CS. The explained variance (**R^2^**) in CS increased from 10.9% to 22.8% when including sex and age as covariates. All path coefficients were significant with p<0.05. The standardised PA pattern, displayed as square root selectivity fraction between the PA intensity variables and the higher order PA construct, indicated the main association in the upper moderate intensity range, decreasing in the vigorous range. The unstandardised PA pattern suggested an association starting in the moderate range, peaking in vigorous intensity and further increasing in the very vigorous range. Since the dependent variable was the same in both models, the PA pattern found to have the highest covariance with the dependent variable was also the same.

Figure 2 presents the third and fourth PLS-SEM models of the association between PA and CRF, with and without covariates. There was a positive association between PA and CRF, which increased slightly when controlling for sex and age. Including sex and age increased the explained variance in CRF from 14% to 39%. All path coefficients were significant with p<0.05. The standardised PA pattern showed the main association from the mid-moderate range and peaked in the upper vigorous range. The unstandardised PA pattern displayed an increasing association with CRF from the mid-moderate range upward, with a minor peak in the vigorous range.

The fifth model regarding the association between CRF and CS, without PA, is not shown in the figures. The path coefficient between CRF and CS was 0.59 (95% CI: 0.56 to 0.61) and the explained variance in CS was 41.6%.

The sixth model, mediating the association between PA and CS through CRF, is shown in figure 3. The association between PA and CRF was almost 10 times stronger than the direct association between PA and CS. The association between CRF and CS remained similar to previous models. The indirect association between PA and CS through CRF was 0.22 (0.20, 0.24) (0.387 * 0.563=0.218) and the total association between PA and CS was 0.27 (0.24, 0.29) (0.218+0.047 = 0.265). Hence, 82% of the association between PA and CS was mediated through CRF (0.218/0.265 = 0.823). Age and sex covariates showed similar associations as in previous models, except for age and CS, which were insignificant. All other path coefficients were significant with p<0.05. The standardised PA pattern showed the main association starting in the mid-moderate intensity range, peaking in the upper part of the moderate intensity, and levelling off slightly in the vigorous range. Like the previous models, the unstandardised PA pattern displayed an increasing association from the mid-moderate range upward, with a small peak in the vigorous range.

The seventh model including an interaction term between sex and CRF and its potential association with CS is not shown in the figures. The path coefficient between the interaction term and CS was insignificant (0.019; 95% CI: −0.005 to 0.044), suggesting no moderation. PA was represented by a higher order composite in all PLS-SEM models, composed of two underlying multi-item composites (PLS components).

## Discussion

The main result of this study is that 82% of the association between PA and CS was mediated through CRF. There were clear associations between PA and CS, PA and CRF and CRF and CS, when not controlling for the other. When including both PA and CRF as independent variables, CRF clearly dominated the association with CS, although there was a weak significant direct association between PA and CS remaining. Hence, there was a partial mediation of the association between PA and CS through CRF, fulfilling the three criteria for mediation.^9^ In a smaller sample, the weak direct association between PA and CS would likely not have been significant and in that case, there would potentially have been a full mediation through CRF. The mediation through CRF suggests that PA has little direct effect on CS, outside of what is explained by the PA that increases CRF.

The novelty of this study in relation to previous research investigating the mediating role of CRF in the association between PA and CS is the detailed analysis of the PA patterns, which revealed differences in what intensities were most influential in the different models. The standardised patterns suggest that moderate intensity explained most of the variation in CS when CRF was not included in the model, whereas vigorous intensity explained most of the variation in CRF. When including both PA and CRF in the model, the PA pattern shared most features from the pattern related to CRF, but with a peak in the moderate range similar to the pattern related to CS. This pattern could be interpreted as the weighted average of the PA patterns related to CRF and CS separately based on the proportion of direct and indirect associations. In addition, the unstandardised PA patterns in all models suggest that 1 min of vigorous intensity PA was consistently more strongly associated with CS compared with 1 min of moderate intensity PA. However, due to the strong multicollinearity between the PA intensity variables and very limited time spent at vigorous intensity, vigorous intensity cannot be interpreted independently from moderate intensity. Hence, according to the PA pattern, 1 min of vigorous intensity would be accompanied by several minutes of moderate intensity.

The difference in PA intensity patterns when including CRF in the models suggests that moderate, and especially vigorous, intensity is important in the association between PA and CRF. However, if methods that are not able to capture vigorous intensity PA accurately are used, the association between PA and CRF would decrease. This could be one explanation for why some previous studies suggest PA and CRF are independent of each other.^17^,^19^ Similar to our results, previous research that have quantified the mediated association between PA and cardiometabolic health through CRF have found a mediation of 73%–93% of the total association.^12 13^ In addition, a larger mediation occurred when vigorous intensity PA was considered.^13^ Furthermore, the strength of the association between PA and cardiometabolic health varies substantially in previous studies, from insignificant to ±0.26, where the strongest associations are similar to this study.^1213 16 19^,^21^ The strongest associations are found in previous studies using objective measures of PA,^12 19^ and measures representing time at MVPA instead of total energy expenditure.^13 21^ Our results suggest that the main association between PA and cardiometabolic health, when including CRF, seems to be in the upper moderate range corresponding to at least 4.5 METs, suggesting that a sufficient intensity of PA is important. Previous research has suggested that this is because 4.5 METs represent moderate intensity relative to the average fitness level in this sample rather than an absolute 3 METs cut-point.^39^ Our research advances this field by employing refined measurement methods and statistical techniques, enabling a detailed exploration of specific PA intensities related to cardiometabolic health, enhancing our comprehension beyond previous studies.

The association between CRF and CS found in this study of 0.59 is higher than in other studies. Still, most studies showed relatively strong associations that ranged from ±0.34 to±0.43,^12 13 16^ except one study that was not able to find a significant association with cardiometabolic health.^21^ This might be due to the use of a submaximal CRF test that assumes a maximal heart rate of 220 subtracted by age, which is a poor estimate of maximal heart rate.^40^

### Limitations

The main limitation of this study is the cross-sectional study design which lacks temporal precedence of the variables studied and hence causation cannot be studied.^9^ To enable proper studying of how CRF mediates the effect of PA, longitudinal measurements are required and the change in CRF and cardiometabolic health, as well as any impact on clinical outcomes such as major adverse cardiovascular events and cardiovascular disease mortality, should be analysed over time. Most previous research using multiple time-points are in line with the present study showing that the mediation through CRF is apparent also in a longitudinal perspective.^10 11 16^ However, one longitudinal study suggest that no mediation through CRF occurs.^21^ The study that found no mediation used total energy expenditure estimated from heart rate measurement as a measure of PA, which might not capture PA intensity. The conflicting results in previous research could therefore be explained by the results of the present study suggesting that PA of a sufficient intensity must be considered.

Furthermore, a mediation of the association between PA and cardiometabolic health through CRF might not be caused by a direct physiological mechanism but could rather represent shared signalling pathways. The physiological pathways related to the association between PA and CRF likely promote pathways related to cardiometabolic health simultaneously, for example, adaptations of the heart, blood vessels and mitochondrial function.^41^ Similarly, CRF and cardiometabolic health seem to share genetic traits, which could partly explain the strong association between the two.^42^ Still, PA seems to be associated with both CRF and cardiometabolic health when controlling for genetics.^43^ In addition, CRF is standardised to body weight which will naturally result in an association with CS since obesity is one of the components. However, the association between CRF and cardiometabolic health remain strong also when controlling for body weight.^44^

Since CRF was only measured in participants without cardiovascular disease, the studied sample displays slightly more favourable cardiometabolic health indicators compared with the general population, which limits the generalisability of the results.^45^ Furthermore, using CS to represent cardiometabolic health is a simplified representation of multiple risk factors. Although CS can predict future cardiometabolic disease well, the association might differ between different risk factors and since the CS is sample-specific, it cannot be directly compared with other populations.^25^

Seven days of accelerometer measurement might be insufficient to capture habitual levels of PA, particularly for vigorous intensity PA.^46^ CRF is more stable over time and could be more representative of habitual MVPA than accelerometer measurements. This could be an alternative explanation to the large mediation of the association between PA and cardiometabolic health through CRF. Longer periods of objectively measured PA, with the ability to accurately capture vigorous intensity PA, are required to investigate this further. Furthermore, the average MVPA level in these results is very high. This is mainly due to the use of short epoch lengths and triaxial data when analysing the accelerometer data.^47^ Therefore, the PA levels cannot be compared with studies using different methods of accelerometer data processing.

Although the PLS-SEM statistical method can handle the collinearity in PA data and enables detailed studying of PA intensity in complex models, the results are difficult to interpret. The results regarding the associations with CS from time spent at specific PA intensities cannot be interpreted independently. Instead, the multivariate PA patterns presented in the figures should be interpreted as a pattern of the associations from all PA intensities together.^23^

### Clinical implications

Our results suggest that most information about the association of PA with cardiometabolic health is captured by a submaximal CRF test. Therefore, PA might be redundant in the analysis of physiological models in epidemiology when CRF measurements are included. CRF itself could be considered a proxy for sufficient volume and intensity of PA for health benefits. However, for the results to be clinically relevant, information about PA is essential since PA is the main varying factor for increasing CRF.^4^

## Conclusions

The results underscore the critical role of PA intensity in interpreting the health benefits of PA, emphasising its importance for a positive impact on health. Accurately capturing and analysing MVPA in detail is essential for comprehensively understanding its effects on cardiometabolic health. Since most of the association between PA and health is mediated through CRF, health-beneficial PA intensity is the intensity sufficient to improve CRF. This fitness-related PA intensity is higher than the PA intensity directly related to cardiometabolic health when not considering CRF. The findings suggest a shift in goals within public health and clinical settings, building on recommendations for PA volume to also emphasising the promotion of more specific activities that enhance CRF.

## supplementary material

10.1136/bjsports-2023-107451 online supplemental file 1

## Data Availability

Data may be obtained from a third party and are not publicly available.
