# Supplementary material for: Fitness-related physical activity intensity explains most of the association between accelerometer data and cardiometabolic health in persons 50–64 years old
Source: Br J Sports Med. 2024 Jul 12;58(21):e107451. doi: 10.1136/bjsports-2023-107451 (PMC11671887; doi:10.1136/bjsports-2023-107451)
Supplement: online supplemental file 1 [file bjsports-58-21-s001.pdf]

Supplemental material

PLS-SEM models set up

The focus of the analyses was not on how well the models fit the data, like in many partial least squares structural equation modelling (PLS-SEM) applications, but instead on the relationship between the variables, like in traditional multiple regression.

To set up the PLS-SEM models, a measurement model and a structural model must be set up.[1] The measurement model refers to what constructs the measured variables represent, and the structural model the relationship between these constructs. Sex, age, fitness and CS were specified as single-item composites in the measurement model. PA was initially specified as one multi-item composite with the variables representing the PA intensity spectrum. To enable multiple latent variables representing PA (PLS components), additional multi-item composites were created. The variables used for the additional composites were generated by deflating the previous PA variables.[2] Deflation was performed by predicting the PA variables from the scores and loadings of the previous composite and subtracting the predicted values from the original values. This removes the variance related to the previous composite and allows for using the deflated PA variables to specify an additional composite. Subsequently, a higher-order composite was specified based on the multiple PA composites. The measurement model of PA is visualized in Supplementary Figure 1. The number of PA composites (PLS components) was selected based on Monte Carlo resampling with 1 000 repetitions and a cut-off of a quarter of a standard deviation, to ensure a model that is significantly better than a model with fewer components.[3]

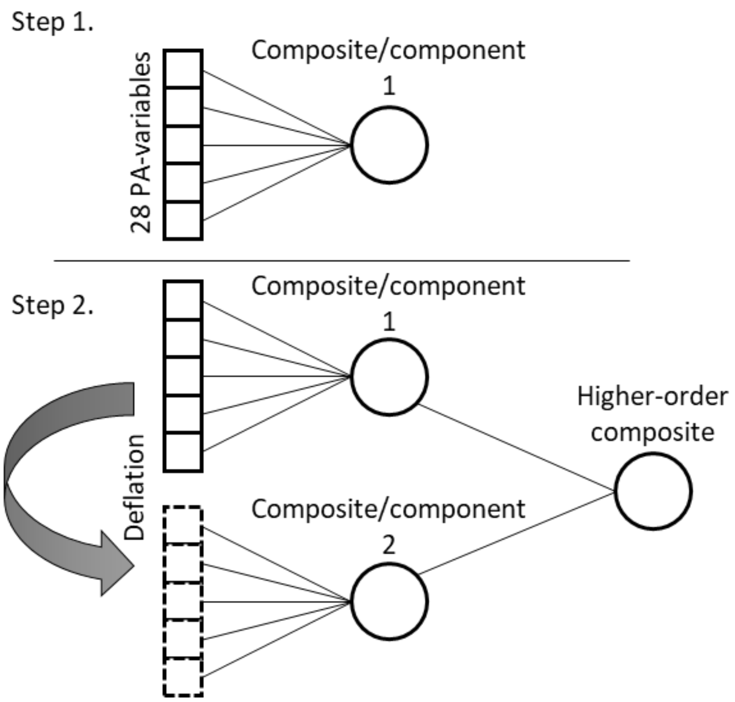

Supplementary Figure 1. Visualisation of the measurement model of the latent composite representing physical activity.

The influence of the different PA intensity variables on the higher-order composite representing PA was indicated by standardised and unstandardised multivariate correlation coefficients of the association between the PA intensity variables and the higher-order PA composite. The multivariate correlation coefficients (standardised) were calculated by taking the square root of the selectivity ratio,[4] which refers to the explained variation in the original PA variables from the latent variables.[5] Subsequently, the multivariate correlation coefficients were unstandardised by dividing by the original variables standard deviation.[4] The standardised multivariate correlation coefficients represent the standardised contribution of the different PA intensities in the higher-order latent variable representing PA. The unstandardised multivariate correlation coefficients represent the contribution of a single minute at the different PA intensities in the higher-order latent variable representing PA. However, the contributions of the specific PA intensity variables are not independent of each other and should be interpreted as an overall pattern that maximises the covariance with the dependent variable.[4] All variables included in the structural model, including the PA latent variable, are independent and interpreted like in traditional multiple linear regression.

Confidence intervals of path coefficients as well as standardised and unstandardised multivariate correlation coefficients were calculated by bootstrapping with 1 000 repetitions. The statistical analyses were performed using R Statistical Software (v4.1.2; R Core Team 2022) and the seminr package (v2.3.2; Ray S, Danks N, Valdez A 2022).[6]

References

1 Hair Jr JF, Hult GTM, Ringle CM, *et al. Partial least squares structural equation modeling (PLS-SEM) using R: A workbook*. Springer Nature 2021.

2 Wold S, Ruhe A, Wold H, *et al.* The Collinearity Problem in Linear Regression. The Partial Least Squares (PLS) Approach to Generalized Inverses. *SIAM J Sci Stat Comput*. 1984;5:735–43.

3 Kvalheim OM, Arneberg R, Grung B, *et al.* Determination of optimum number of components in partial least squares regression from distributions of the root-mean-squared error obtained by Monte Carlo resampling. *J Chemom*. 2018;32:e2993.

4 Aadland E, Andersen LB, Resaland GK, *et al.* Interpretation of Multivariate Association Patterns between Multicollinear Physical Activity Accelerometry Data and Cardiometabolic Health in Children—A Tutorial. *Metabolites*. 2019;9:129. doi: 10.3390/metabo9070129

5 Kvalheim OM. Interpretation of partial least squares regression models by means of target projection and selectivity ratio plots. *J Chemom*. 2010;24:496–504.

6 Ray S, Danks NP, Valdez AC. *seminr: Building and Estimating Structural Equation Models*. 2022. https://CRAN.R-project.org/package=seminr
